# Absent in melanoma 2 (AIM2) in rheumatoid arthritis: novel molecular insights and implications

**DOI:** 10.1186/s11658-022-00402-z

**Published:** 2022-12-07

**Authors:** Jianan Zhao, Shicheng Guo, Steven J. Schrodi, Dongyi He

**Affiliations:** 1 Department of Rheumatology, Shanghai Guanghua Hospital, Shanghai University of Traditional Chinese Medicine, Shanghai, China; 2 Guanghua Clinical Medical College, Shanghai University of Traditional Chinese Medicine, Shanghai, China; 3 Computation and Informatics in Biology and Medicine, University of Wisconsin-Madison, Madison, WI USA; 4 Department of Medical Genetics, School of Medicine and Public Health, University of Wisconsin-Madison, Madison, WI USA; 5 Arthritis Institute of Integrated Traditional and Western Medicine, Shanghai Chinese Medicine Research Institute, Shanghai, China; 6 Institute of Arthritis Research in Integrative Medicine, Shanghai Academy of Traditional Chinese Medicine, Shanghai, China

**Keywords:** Rheumatoid arthritis, Autoimmune disease, Absent in melanoma 2, Inflammation, Pyroptosis, PANoptosis

## Abstract

Absent in melanoma 2 (AIM2), a member of the Pyrin and HIN domain protein family, is a cytoplasmic receptor that recognizes double-stranded DNA. AIM2 exhibits limited expression under physiological conditions but is widely expressed in many human diseases, including autoimmune diseases, and plays an essential role in the immune response. Rheumatoid arthritis (RA) is an autoimmune disease that poses a severe threat to physical and mental health, and is caused by several genetic and metabolic factors. Multiple immune cells interact to form a complex inflammatory network that mediates inflammatory responses and bone destruction. Abnormal AIM2 expression in multiple immune cell populations (T cells, B cells, fibroblast-like synoviocytes, monocytes, and macrophages) may regulate multiple functional responses in RA through mechanisms such as pyroptosis, PANoptosis, and regulation of other molecules. In this review, we describe and summarize the functional regulation and impact of AIM2 expression in immune cells to improve our understanding of the complex pathological mechanisms. These insights may provide potential directions for the development of new clinical diagnostic strategies for RA.

## Introduction

Rheumatoid arthritis (RA) is a heterogeneous autoimmune disease characterized by chronic synovial inflammation and the destruction of bones and joints. RA can be classified as anti-citrullinated protein antibody (ACPA)-negative or ACPA-positive, based on the autoantibody profile [1]. RA affects 0.5–1% of the global population, with women being more likely to be affected [2]. In addition to the classic manifestations of joint destruction, RA often affects the skin, liver, kidneys, heart, and other organs [2]. The main pathological mechanisms of RA involve interactions among genetic, environmental, metabolic, immune, and microbial flora, cell death, and others [3, 4]. Currently, several treatment options are available for RA; first-line management includes nonsteroidal anti-inflammatory drugs, corticosteroids, and opioid analgesics. Second-line management includes disease-modifying antirheumatic drugs, such as methotrexate and hydroxychloroquine. However, a range of biological agents, such as tumor necrosis factor-alpha (TNF-α) inhibitors, have also been developed. Patients with RA respond differently to therapy owing to the complex pathological mechanisms, disease heterogeneity, and drug side effects. This can significantly affect patients’ physical and mental health, as well as the clinical outcomes. Therefore, elucidating the biomolecular mechanisms of the disease, identifying new therapeutic targets, and developing innovative clinical treatment options is crucial.

Melanoma 2 (AIM2) is a member of the interferon (IFN)-induced pyrin and HIN domain family of proteins, which has three other members in humans—IFN-inducible protein 16, nuclear localization (HIN) domain family member 1, and myeloid cell nuclear differentiation antigen—and 12 other members in mice, including myeloid cell nuclear differentiation antigen and myeloid nuclear differentiation antigen-like[5]. AIM2 activation is reduced under physiological conditions. AIM2 was identified as a cytoplasmic deoxyribonucleic acid (DNA) sensor by an orthogonal proteomic–genomic screen and exhibits specificity for double-stranded DNA [6]. By recognizing microbial DNA from various sources, AIM2 may act as a defense mechanism, preventing pathogens, mainly bacteria and viruses, from infecting the organism. Lugrin et al. reviewed a variety of bacteria and viruses that can be recognized by AIM2 [7]. Abnormal deletion and dysregulation of AIM2 is thought to be associated with a variety of diseases, including autoimmune diseases and cancer [8]. RA, which involves various immune cells, is characterized by the abnormal proliferation of fibroblast-like synoviocytes (FLS), the presence of autoimmune T and B cells, and an increase in the levels of proinflammatory macrophages. AIM2 can affect different cell subpopulations and inflammatory responses through pyroptosis and PANoptosis. However, AIM2 is also regulated by other mechanisms that mediate abnormal responses in RA. AIM2 inhibition may be a strategy for treating RA. Defective DNA processing in a mouse model of deoxyribonuclease (DNase) II deficiency causes the ectopic transfer of DNA to the cytoplasm, where it accumulates, activating the stimulator of interferon gene (STING) and increasing the production of type I IFN and proinflammatory cytokines [9]. Therefore, mouse models of DNase II deficiency or dual DNase II/interferon-α/β receptor (IFNAR) deficiency exhibit symptoms similar to those observed in patients with arthritis, accompanied by the production of multiple cytokines such as TNF-α, interleukin(IL)-6, and IL-1β [10]. Reduced joint inflammation in AIM2/DNase II dual-deficient mice is accompanied by reduced inflammatory vesicle activation and decreased caspase-1 and IL-1β production [9]. AIM2 inflammasome activation in DNase II/IFNAR/AIM2 triple-knockout arthritic mice is limited, reducing joint inflammation, IL-18 protein expression in the joints, and systemic IL-18 levels [11]. In this review, we summarize the mechanism of AIM2 in RA to define the relationship between AIM2 and RA, and provide a basis for the future development of individualized treatments.

### AIM2 inflammasome-mediated pyroptosis is associated with RA

Pyroptosis is the death of proinflammatory cells. Classical pyroptosis, induced by the nucleotide oligomerization domain-like receptor family pyrin domain-containing 3(NLRP3) inflammasome, is associated with RA [12]. The AIM2 inflammasome may have originated as an essential response to clear microbes and damaged cells [13]. The structure and assembly processes have been extensively described; in short, AIM2 is a cytoplasmic sensor that recognizes double-stranded DNA of both intra- and extracellular origin. The C-terminal hematopoietic expression, interferon-inducible nature, and nuclear localization (HIN) domains of AIM2 are responsible for the nonspecific, length-dependent recognition and binding of double-stranded DNA, which bring AIM2 out of its resting inhibitory state and promote oligomerization. The N-terminal pyrin domain (PYD) of AIM2 is primarily responsible for the recruitment and binding of the PYD of pyrin and caspase recruitment domain-containing (ASC), which promotes the recruitment of procaspase-1 and the formation of the AIM2 inflammasome [14–17]. AIM2 inflammasome formation activates caspase-1 downstream and promotes the maturation of pro-1β and pro–IL-18 to IL-1β and IL-18, respectively, which are then released into the extracellular environment through pyroptosis [13]. The elevated levels of IL-1β and IL-18 in both the serum and synovial fluid of patients with RA are thought to be characteristic of inflammation [18]. IL-18 is associated with RA via multiple mechanisms [19]. In joints, IL-18 interacts with endothelial cells, FLS, monocytes, and neutrophils to promote inflammation by upregulating the expression of cell adhesion factors and chemokines; however, IL-18 can also induce angiogenesis [19]. AIM2 inflammasome assembly may require multiple mechanisms. For example, cellular Fas-associated death domain-like interleukin-1β-converting enzyme (FLICE)-inhibitory protein interacts with procaspase-1 and is essential in AIM2 inflammasome assembly and downstream mediator activation. Inhibition of cellular FLICE-inhibitory protein reduces AIM2 activation and inhibits IL-1β production via a noncanonical caspase-8-mediated pathway [20]. The BH3 structural domain of the homologous to E6AP C-terminus (HECT), ubiquitin-associated domain (UBA), and the Trp–Trp–Glu (WWE) domain containing ubiquitin-protein ligase (E3) ubiquitin ligase 1 (HUWE1) binds to the HIN structural domain of AIM2 and mediates the K27-linked polyubiquitination of AIM2, leading to inflammasome assembly. Inhibition of HUWE1 significantly reduces inflammasome assembly and its downstream effects [21]. In conclusion, RA may be affected by AIM2-mediated pyroptosis in different cell subpopulations.

### AIM2-mediated PANoptosis is associated with RA

PANoptosis has been characterized in several studies. PANoptosis involves PANoptosomes, which comediate inflammatory PANoptosis through the interaction of three forms of cell death: apoptosis, pyroptosis, and necroptosis. Different PANoptosomes share common proteins, such as receptor-interacting serine/threonine-protein kinase (RIPK)1, RIPK3, caspase-1, and caspase-8. However, there is some variation among PANoptosomes in terms of their sensors, such as Z-DNA binding protein 1 (ZBP1), AIM2, NLRP3, and NLR family caspase recruitment domain-containing 4. Downstream, PANoptosomes share common effector molecules for apoptosis (caspase-3 and -7), necroptosis [mixed lineage kinase domain-like pseudokinase (MLKL)], and pyroptosis [gasdermin D (GSDMD)], which synergistically activate PANoptosis [22]. For example, caspase-6 interacts with RIPK3 to promote the binding of RIPK3 to ZBP1 in response to influenza A infection. Caspase-6 also recruits RIPK1 and caspase-8 to form a PANoptosome [23, 24]. Furthermore, macrophages responding to infection with influenza A virus, vesicular stomatitis virus, *Listeria monocytogenes*, and *Salmonella enterica* serovar Typhimurium trigger strong ZBP1-mediated PANoptosis. Notably, single-molecule inhibition of PANoptosis does not occur, whereas combined inhibition by multiple molecules (caspase-1, RIPK3, and caspase-8) can inhibit PANoptosis [25].

**Fig. 1:**
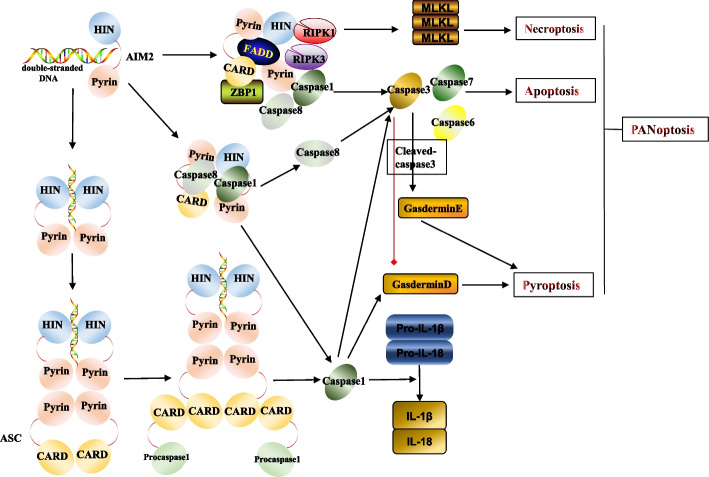
Absent in melanoma 2 (AIM2)-mediated pyroptosis and PANoptosis. AIM2 assembles into the AIM2 inflammasome by recognizing double-stranded DNA and subsequently oligomerizing and binding ASC and procaspase-1. The cleavage of procaspase-1 promotes the maturation of caspase-1. Caspase-1, in turn, promotes the maturation of pro-1β and pro-18 and cleaves gasdermin D (GSDMD) to promote the disruption of cell membranes and the release of IL-1β and IL-18. AIM2 also binds ASC, FADD, RIPK1, RIPK3, caspase-1, caspase-8, and ZBP1 to assemble the PANoptosome and activate downstream effectors, including the apoptosis effectors (caspase-3, -6, and -7), pyroptosis effector (GSDMD), and necroptosis effector (MLKL), thereby promoting PANoptosis. In addition, AIM2 binds caspase-8, caspase-1, and ASC to form a complex, activating caspase-8 and caspase-1, cleaving downstream caspase-3, and ultimately promoting apoptosis. Cleaved caspase-3 inhibits gasdermin D and mediates pyroptosis through gasdermin E. Caspase-1 also activates pyroptosis via gasdermin

AIM2 binds ASC, caspase-8, and caspase-1 to form an inflammatory complex, activating caspase-8 and caspase-1 and leading to the cleavage of caspase-3. Cleaved caspase-3 triggers apoptosis through poly (adenosine diphosphate ribose) polymerase-1, whereas caspase-1 activates pyroptosis through GSDMD family proteins [26]. Further studies have demonstrated that AIM2 binds pyrin, ZBP1, ASC, caspase-1, caspase-8, RIPK1, RIPK3, and Fas-associated death domain (FADD) to form a PANoptosome and activate PANoptosis [27]. Multiple forms of cell death, including apoptosis, pyroptosis, and necroptosis, are observed in RA [12]. The apoptotic suppression of FLS and autoimmune cells contributes to their abnormal proliferation, whereas excessive apoptosis of osteoblasts promotes bone destruction. Pyroptosis and necroptosis contribute to inflammation in various cells [12]. PANoptosis, as a form of crosstalk involving apoptosis, pyroptosis, and necroptosis, may have a potential role in RA. Notably, critical molecules of pyroptosis and necroptosis are upregulated in several cell populations in RA. For example, the expression of RIPK1, RIPK3, and p-MLKL was increased in an experimental animal model of arthritis in vivo and in acid-induced chondrocytes in vitro [28, 29]. Compared with controls, monocytes from patients with RA exhibit increased expression of the *ASC*, *NLRP3 full-length*,and caspase1(*CASP1*) genes; serum levels of caspase-1 and IL-18 are also increased [30]. These characteristics may contribute to PANoptosis in a variety of cells, leading to inflammation. This promising research direction deserves further exploration, given the limited research on AIM2-mediated PANoptosis and RA (Fig. 1).

### AIM2 engages in the functional regulation of T cell subsets affecting RA

Risk factors for RA include abnormal metabolism in T cell subpopulations and excessive T cell apoptosis owing to defective 6-phosphofructo-2-kinase/fructose-2,6-biphosphatase 3 expression [31]. The release of intracellular DNA after tissue injury activates AIM2 inflammasome assembly and IL-1β release in myeloid cells. This promotes Fas ligand expression in monocytes, which binds to Fas in T cells and promotes apoptosis through the FADD/caspase-8 pathway [32]. Therefore, activation of AIM2 may promote RA by stimulating excessive apoptosis in T cells.

Abl proto-oncogene 1, a nonreceptor tyrosine kinase, influences the inflammatory response in autoimmune T cell development, possibly regulating AIM2. ABL proto-oncogene 1 is a vital T cell development factor that responds to DNA damage. It promotes the phosphorylation of IFN regulatory factor 3 (IRF3) and enhances TANK-binding kinase 1 (TBK1)-dependent IRF3 activation, leading to increased IRF3 transcriptional activity and IFN-β expression [33]. IFN-β is a crucial factor in AIM2 activation and may further promote the activation and downstream effects of AIM2 [34]. The functional defects of Treg cells in RA, which prevent the effective suppression of autoimmune T cell responses, may partly be due to defective AIM2 expression. In humans and mice, normal Treg cells highly express AIM2 and are regulated by a series of Treg cell transcription factors, such as *runt-related transcription factor 1*, E 26 (*ETS*) *proto-oncogene 1*, *B-cell leukemia 11b*, and *cyclic adenosine monophosphate (cAMP)-response element-binding protein* [35]. AIM2 inhibits protein kinase B (AKT) phosphorylation, mammalian target of rapamycin (mTOR), c-Myc, and glycolysis, but promotes oxidized lipid phosphorylation in Treg cells. Mechanistically, AIM2 interacts with the receptor for the activated C kinase 1/protein phosphatase 2 phosphatase complex to inhibit AKT phosphorylation. AIM2 also promotes Treg stabilization during inflammation. Thus, AIM2 plays an important role in suppressing T cells involved in autoimmunity by reducing AKT-mTOR signaling and altering immune metabolism, which enhances Treg cell stability [35]. In addition, noncoding ribonucleic acid (RNA)s are closely associated with RA [36, 37], and a Treg/ T helper 17 (Th17) cell imbalance is a common pathological factor in RA [3]. Long-stranded noncoding RNA nuclear enriched abundant transcript 1 (*NEAT1*) affects the balance between Treg/Th17 cells by inhibiting AIM2 through the microRNA(miR)-485-5p sponge. A decrease in the number of Treg cells is negatively correlated with *NEAT1* expression, whereas an increase in the number of Th17 cells is positively correlated with *NEAT1* expression [38].

### AIM2 participates in the functional regulation of B cell subsets, promoting inflammation

The anti-inflammatory factor IL-10 is mainly produced in the cluster of differentiation (CD)19 + CD27 + memory B cell subset. These cells are significantly reduced in number in patients with RA and do not suppress the production of IFN-γ by CD4 + T cells [39]. Correspondingly, patients with RA exhibit a significantly increased subpopulation of immunoglobulin D(IgD)-CD27-B cells, which can be reduced by treatment with TNF inhibitors and tolimumab [40]. Stable, high expression of AIM2 was observed in CD27 + B cells of peripheral blood; these cells release IL-1β in response to in vitro DNA stimulation [41]. Therefore, the reduction in the CD19 + CD27 + memory B cell subpopulation may occur as a result of cell-derived DNA-activated AIM2-mediated cell death in RA, ultimately promoting inflammation. Notably, rituximab may induce apoptosis in peripheral blood IgD-CD27 + and IgD + CD27 + B cell subsets, which may be one of the reasons for the poor response to rituximab in some patients with RA [42]. The B cell lymphoma 6 (BCL6)-B-lymphocyte-induced maturation protein 1 (BLIMP1) axis is associated with receptor activator of nuclear factor k-B ligand (RANKL)-induced osteoclast differentiation and bone destruction in RA [43]. B cells from patients with systemic lupus erythematosus express AIM2 and promote B cell differentiation by regulating the BCL6–BLIMP1 axis [44]. Therefore, in view of the above results, AIM2 may act as an upstream regulator that possibly affects B cell differentiation by decreasing BLIMP1 expression and increasing BCL6 expression. AIM2 may also affect osteoblast differentiation by regulating the BCL6–BLIMP1 axis. The connection among AIM2, BCL6, and BLIMP1 requires further exploration in the context of RA.

### AIM2 is involved in the functional regulation of FLS and monocytes, leading to cellular over-survival and inflammation

FLS exist in an abnormal proliferative state, which leads to the excessive release of inflammatory mediators and causes bone destruction and angiogenesis in patients with RA [45, 46]. High expression of AIM2 may be involved in abnormal proliferative processes and inflammatory responses in the synovium. First, *AIM2* was identified as a differentially expressed gene in RA synovial tissue, and enrichment analysis revealed that *AIM2* is involved in “immune response” and “inflammatory response” processes [47]. The interaction between AIM2, Jun kinase, and glutathione peroxydases (GPx) may be involved in the pathology of RA, including regulation of angiogenesis, inflammation, and synovial cell proliferation [48–52]. AIM2 levels are significantly increased in the sera of patients with RA. The expression of AIM2, ASC, caspase-1, and IL-1β in synovial tissue is increased, and the levels of AIM2, ASC, and IL-1β are positively correlated with the erythrocyte sedimentation rate and C-reactive protein levels in patients [53]. Second, transforming growth factor-β-activated kinase 1 (TAK1) acts as a negative regulator of PANoptosis [25]. TAK1 expression was upregulated in the FLS of both RA and collagen-induced arthritis (CIA) mice [54]. The long noncoding RNA (lncRNA) *linc00152* in RA FLS represses miR-103a, promoting the upregulation of TAK1 and the activation of the nuclear factor kappa-light-chain enhancer of activated B cells (NF-κB) pathway, ultimately inducing TNF-α and IL-1β expression [55]. Therefore, TAK1 upregulation in RA FLS may cause abnormal cell proliferation and inflammatory responses by inhibiting PANoptosis. Finally, the addition of AIM2-small interfering RNA significantly reduces the proliferation of RA FLS, demonstrating that AIM2 inhibition could potentially improve RA [53].

RA-related atherosclerosis is a serious cardiovascular complication. Oxidation of low-density lipoproteins (OX-LDL) is a risk factor for RA, promoting the release of matrix metallopeptidase (MMP)-1 and MMP-3 from RA FLS [56]. OX-LDL and levels of soluble lectin-like oxidized low-density lipoprotein receptor 1 (sLOX-1) are elevated in the plasma and synovial fluid of patients with RA [56, 57], and sLOX-1 levels are positively correlated with disease activity in RA [56]. In a mouse model of arthritis, inhibition of the OX-LDL/LOX-1 interaction improved symptoms and reduced MMP-1 and MMP-3 production [56, 58]. Elevated serum levels of OX-LDL in CIA mice may promote RA-related atherosclerosis progression [59]. Studies have shown that AIM2 and GSDMD-N expression show a concentration-dependent relationship with OX-LDL levels [60]. Thus, AIM2, GSDMD-N, and OX-LDL may synergistically affect RA-associated atherosclerosis.

An earlier study reported that the number of CD14 + AIM2 + monocytes decreased in patients with RA. Monocytes from patients with RA exhibit increased levels of IL-1β before and after lipopolysaccharide (LPS) stimulation in vitro, although possibly independent of AIM2 signaling. Increased expression of the mTOR-associated protein LST8 homolog (POP3) in monocytes inhibits AIM2 expression, promoting monocyte survival and differentiation to proinflammatory cells, thereby exacerbating the inflammatory process [61].

### AIM2 is involved in the functional regulation of macrophages affecting inflammation

Multiple cell populations exhibit increased differentiation and proliferation in response to numerous inflammatory mediators, and proinflammatory macrophages are essential effector cells of inflammation in RA. The increased expression of AIM2 positive regulators and decreased expression of AIM2 negative regulators in the RA environment promotes inflammatory responses through AIM2 expression in macrophages. The primary positive regulators of AIM2 in RA macrophages include cholesterol, phosphoglycerate mutase family member 5 (PGAM5), nuclear factor E2-related factor-2 (Nrf2), the lncRNA *NEAT1*, and high-mobility group box-1 (HMGB1).

Cell differentiation and proliferation require high cholesterol levels to promote cell membrane synthesis [62]. In macrophages, elevated cholesterol levels activate the AIM2 inflammasome and induce the release of IL-1β by mechanisms that may involve cholesterol-25-hydroxylase downregulation, cholesterol synthesis pathway activation, mitochondrial dysfunction, and mitochondrial DNA release [62]. PGAM5 regulates mitochondrial function and reactive oxygen species production in macrophages by regulating AIM2 inflammasome activation and downstream caspase-1 expression to promote ASC recruitment and inflammatory responses [63]. Nrf2 is a classic antioxidative stress molecule that inhibits the proliferation and migration of RA FLS. Inflammatory factors attenuate the oxidative stress response by binding to antioxidant components [64]. However, Nrf2 is an important factor in activating the AIM2 inflammasome in macrophages. Nrf2-knockout macrophages exhibit reduced levels of the AIM2 inflammasome, caspase-1, and IL-1β [65]. Therefore, the link between Nrf2 and AIM2 requires further investigation and may be cell type dependent. Over-proliferation of FLS causes a hypoxic microenvironment in RA synovial tissue. In mouse macrophages, *NEAT1* upregulates hypoxia-inducible factor 2α in the hypoxic microenvironment, leading to AIM2 inflammasome assembly and the release of caspase-1, IL-1β, and IL-18, which promotes inflammation [66]. HMGB1 can be released extracellularly in response to different stimuli or from necrosis-like cells. It binds IL-1α, IL-1β, and LPS to form complexes and activates advanced glycosylation end-product-specific receptors (RAGE), toll-like receptors (TLRs), and other receptor ligands to promote downstream inflammatory processes. HMGB1 is considered a promising therapeutic target for RA [67]. HMGB1 promotes proinflammatory M1 macrophage polarization by inducing inflammation via activation of the AIM2, TLR2, TLR4, and RAGE/NF-κB signaling pathways [68]. Inhibition of pyruvate kinase muscle (PKM2)/eukaryotic translation initiation factor 2 alpha kinase 2 (EIF2AK2) attenuates AIM2 activation and the release of IL-1β, IL-18, and HMGB1, which can suppress inflammatory responses. PKM2 promotes AIM2 activation by regulating macrophage glycolysis and affecting EIF2AK2 phosphorylation [69].

In macrophages, PKM2/EIF2AK2 is a negative regulator of AIM2. Negative regulators of AIM2 also include bilirubin, high-temperature requirement protein A2 (HtrA2), and the omega-3 fatty acid docosahexaenoic acid. Bilirubin, an antioxidant and immunomodulator, is a potential protective factor against RA [70, 71]. Serum bilirubin levels in patients with RA are reduced and negatively correlated with the levels of disease activity and inflammatory markers, which include the erythrocyte sedimentation rate and C-reactive protein [70, 72]. In macrophages, bilirubin inhibits AIM2 inflammasome assembly, reduces the phosphorylation of nuclear factor of kappa light polypeptide gene enhancer in B-cells inhibitor alpha (IκB-α), and inhibits p65-mediated suppression of LPS-induced TNF-α and IL-6 secretion, thereby suppressing inflammation [73]. HtrA2 has a variety of ameliorative effects on RA, including inhibition of AIM2. For example, in CIA mice, HtrA2 induces signal transducer and activator of transcription 3 cleavage by inhibiting Th17 cell differentiation and improving arthritic symptoms [74]. Defective HtrA2 in CIA mice leads to reduced TNF receptor-associated factor 2 stability, promoting the production of proinflammatory factors by macrophages [75]. HtrA2 reduces ASC recruitment and regulates autophagy to inhibit AIM2-mediated inflammatory responses in macrophages [76]. Similarly, docosahexaenoic acid limits macrophage inflammatory responses by inhibiting nuclear translocation of NF-kB, reducing AIM2 inflammasome assembly, and promoting autophagy by reducing IL-1β levels [77]. AIM2-mediated pyroptosis and autophagy may be antagonistic processes in RA macrophages. Studies have demonstrated that the intake of omega-3 polyunsaturated fatty acids significantly improves the levels of disease activity markers in patients with RA [78]. In macrophages, AIM2 inflammasomes can activate the G protein ras-like proto-oncogene B(RalB) and promote autophagosome formation during autophagy through inflammasome sensors. Autophagosomes prevent destructive inflammatory responses by the uptake of AIM2 inflammasomes through the autophagic bridging subunit P62; this mechanism may inhibit AIM2-mediated inflammation in RA [79].

Notably, cellular DNA is sensed by both AIM2 and cyclic GMP-AMP synthase (cGAS), leading to downstream pyroptosis and type I IFN responses, respectively. GSDMD, a key effector molecule of pyroptosis, inhibits the cGAS-driven type I IFN response by promoting potassium efflux through pore formation in the macrophage membrane [80]. Caspase-1 can also inhibit STING activation by cleaving cGAS [81]. Excess TNF activates the cGAS/STING pathway of cytoplasmic DNA to induce a type I IFN response and promote inflammation. cGAS deficiency in CIA mice prevents inflammatory cell infiltration and reduces joint swelling [82]. Therefore, macrophages appear to promote inflammatory responses primarily through the upregulation of AIM2, rather than through the cGAS/STING pathway.

## Conclusions

**Fig. 2:**
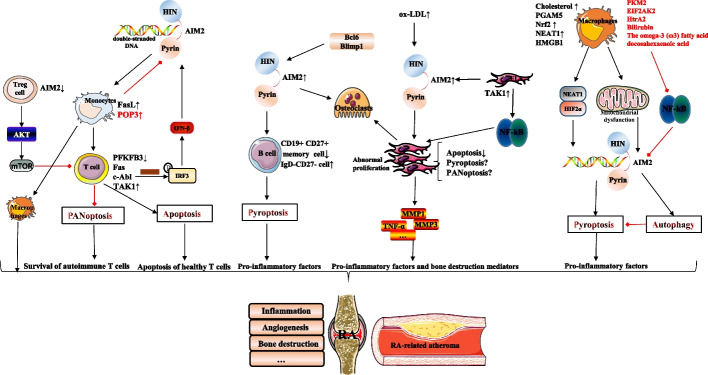
AIM2 regulatory network of multiple cell subpopulations in rheumatoid arthritis (RA). Aberrant expression of AIM2 in various cells in RA promotes different manifestations of RA, including bone destruction, angiogenesis, inflammation, and RA-associated atherosclerosis. For example, upregulation of AIM2 expression promotes excessive apoptosis in healthy T cells, and overexpression of TAK1 may inhibit PANoptosis in autoimmune T cells and FLS to promote self-survival. The BCL6/BLIMP1 axis regulates osteoclast and B cell differentiation via AIM2. The upregulation of AIM2 in FLS promotes cell proliferation and the subsequent release of inflammatory factors and bone destruction mediators. The upregulation of POP3 in monocytes suppresses AIM2 expression and promotes monocyte survival and differentiation into proinflammatory macrophages. The presence of AIM2 positive regulators (black) and AIM2 negative regulators (red) in macrophages affects macrophages and their downstream inflammatory processes through different mechanisms

RA is a severe chronic inflammatory disease that may lead to disability and seriously affect the physical and mental health of patients if not effectively treated. Early detection and intervention are necessary to prevent progression. Applying effective clinical protocols to patients in the mid-stage of RA is also essential to avoid serious damage. However, clinical management remains a huge challenge owing to multiple factors, such as the varying clinical responses of patients to numerous therapies. Therefore, there is an urgent need to elucidate the mechanisms of the disease and develop innovative and effective clinical treatment options. AIM2 is essential for the inflammatory response in RA, as it plays a critical role in pyroptosis and PANoptosis. AIM2 expression is involved in other RA mechanisms and in the abnormal function of different cell subpopulations. A number of targeted AIM2 inhibitors and antagonists exist, such as J114 [83], synthetic oligodeoxynucleotides including the immunosuppressive motif TTAGGG [84], and shikonin [85]. The therapeutic effect of AIM2 inhibitors against RA remains unknown; especially, the effect on AIM2-mediated PANoptosis is worth exploring. We summarize the potential effects of AIM2 inhibition on RA (Table 1 and Fig. 2). There are still some questions that remain unanswered. AIM2 in normal cells is generally localized in the cytoplasm, and the nuclear membrane acts as a physical barrier to prevent AIM2 from sensing self-DNA. The existence of additional mechanisms involved in RA that facilitate the sensing of self-DNA by AIM2 remains unknown. Additionally, never in mitosis gene a-related kinase 7 (NEK7), a vital factor in NLRP3 inflammasome-activated pyroptosis, is thought to prevent the excessive activation of NLRP3 inflammasomes. The excessive activation of AIM2 in RA may result from a disruption in a similar self-protection mechanism. This hypothesis is of great clinical significance for the in-depth determination of the complex pathological mechanisms of RA and the development of innovative treatments.

**Table 1:** Potential effects of absent in melanoma 2 (AIM2) inhibition on RA

Cell populations	The potential effects of inhibition of AIM2	References
T-cell subsets	AIM2 inhibition may alleviate rheumatoid arthritis (RA) by suppressing excessive apoptosis of T cells	[31, 32]
	AIM2 inhibition may inhibit autoimmune T-cell development and function, suppressing excessive inflammatory responses	[33, 34]
	AIM2 inhibition in Treg cells may lead to their functional defects. In addition, inhibition of AIM2 by some microRNA long noncoding RNA interactions may affect the balance between Treg/ T helper 17 (Th17) cells	[35, 38]
B-cell subsets	AIM2 inhibition of the cluster of differentiation(CD)27 + B cells may promote the release of the anti-inflammatory factor IL-10 and inhibit pyroptosis from suppressing inflammation	[39, 41, 42]
	AIM2 inhibition may affect B-cell differentiation by regulating The B cell lymphoma 6 (BCL6)-B-lymphocyte-induced maturation protein 1 (BLIMP1) expression	[44]
Fibroblast-like synoviocytes (FLS)	AIM2 inhibition may inhibit FLS hyperproliferation and inflammatory responses. In addition, AIM2 inhibition may affect RA-associated atherosclerosis by regulating oxidized low-density lipoprotein (ox-LDL) and gasdermin D (GSDMD)-N	[53, 60]
Monocytes	AIM2 inhibition in monocytes may promote their survival and facilitate differentiation to pro-inflammatory cells, thereby exacerbating the inflammatory process	[61]
Macrophages	AIM2 inhibition in macrophages reduces the activation of pyroptosis and promotes autophagy to reduce the inflammatory response	[63, 79]

## Data Availability

Not applicable.

## Abbreviations

AIM2: Absent in melanoma 2
RA: Rheumatoid arthritis
ACPA: Anti-citrullinated protein antibody
TNF-α: Tumor necrosis factor-α
IFN: Interferon
HIN: Hematopoietic, interferon inducible, and nuclear
DNA: Deoxyribonucleic acid (DNA)
FLS: Fibroblast-like synoviocytes
DNase: Deoxyribonuclease
STING: Stimulator of interferon gene
IFNAR: Interferon-α/β receptor
IL-6: Interleukin-6
NLRP3: The nucleotide oligomerization domain-like receptor family pyrin domain-containing 3
PYD: Pyrin domain
ASC: PYD and CARD domain containing
FLICE: Fas-associated death domain-like interleukin-1β-converting enzyme
HECT: Homologous to E6AP C-terminus
UBA: Ubiquitin-associated domain
WWE: Trp–Trp–Glu
E3: Ubiquitin-protein ligase
HUWE1: Homologous to E6AP C-terminus (HECT), ubiquitin-associated domain (UBA), and the Trp–Trp–Glu (WWE) domain containing ubiquitin-protein ligase (E3) ubiquitin ligase 1
RIPK: Receptor-interacting serine/threonine-protein kinase
ZBP-1: Z-DNA binding protein 1
MLKL: Mixed-lineage kinase domain-like pseudokinase
GSDMD: Gasdermin D
FADD: Fas-associated death domain
IRF3: IFN regulatory factor 3
TBK1: TANK-binding kinase 1
ETS: E 26
Camp: Cyclic adenosine monophosphate
mTOR: Mammalian target of rapamycin
Th17: T helper 17
RNA: Ribonucleic acid
NEAT1: Nuclear-enriched abundant transcript 1
miR: MicroRNA
CD19: Cluster of differentiation 19
IgD: Immunoglobulin D
BCL6: B cell lymphoma 6
BLIMP1: B-lymphocyte-induced maturation protein 1
RANKL: Receptor activator of nuclear factor k-B ligand
GPx: Glutathione peroxidase
TAK1: Transforming growth factor-β-activated kinase 1
lncRNA: Long non-coding RNA
LPS: Lipopolysaccharide
NF-κB: Nuclear factor kappa-light-chain enhancer of activated B cells
OX-LDL: Oxidation of low lipoprotein
MMP: Matrix metallopeptidase
sLOX-1: Soluble lectin-like oxidized low-density lipoprotein receptor 1
CIA: Collagen-induced arthritis
LPS: Lipopolysaccharide
POP3: MTOR-associated protein
PGAM5: Phosphoglycerate mutase family member 5
Nrf2: Nuclear factor E2-related factor-2
HMGB1: High-mobility group box-1
RAGE: Advanced glycosylation end-product-specific receptors
TLR: Toll-like receptors
PKM2: Pyruvate kinase muscle
EIF2AK2: Eukaryotic translation initiation factor 2 alpha kinase 2
HtrA2: High-temperature requirement protein A2
IκB-α: Inhibitor alpha
RalB: Ras-like proto-oncogene B
cGAS: Cyclic GMP–AMP synthase
NEK7: Never in mitosis gene a-related kinase 7
